# Parental practices and their association with alcohol and cannabis use among adolescents in Chile

**DOI:** 10.3389/fpsyg.2023.1209584

**Published:** 2023-09-11

**Authors:** Nicolás Libuy, Viviana Guajardo, Carlos Ibáñez, Ana María Araneda, Lorena Contreras, Paula Donoso, Jorge Gaete, Adrian P. Mundt

**Affiliations:** ^1^Department of Psychiatry and Mental Health, Medical Faculty, Universidad de Chile, Santiago, Chile; ^2^Doctorado en Psicoterapia, Facultad de Medicina y Facultad de Ciencias Sociales, Universidad de Chile y Universidad Católica de Chile, Santiago, Chile; ^3^ANID, Millennium Science Initiative Program, Millennium Nucleus to Improve the Mental Health of Adolescents and Youths, Imhay, Santiago, Chile; ^4^Research Center for School Mental Health, Faculty of Education (ISME), Universidad de Los Andes, Santiago, Chile; ^5^Medical Faculty, Universidad Diego Portales, Santiago, Chile

**Keywords:** parenting, school survey, adolescence, cannabis, alcohol, substance use, prevention

## Abstract

**Background:**

Adolescent alcohol and cannabis use are common in Chile. The present study aimed to assess the relationship between perceived parenting practices and alcohol and cannabis use among adolescents in a Latin American context.

**Methods:**

We adapted and implemented a substance use prevention strategy in Chile, which included surveys of tenth-grade students from six municipalities in the Metropolitan Region of Greater Santiago. We assessed the reliability and factorial structure of the parenting scale with 16 items, which formed part of the survey. We dichotomized parenting scores into high (above the median) and low. The association of parenting practices with alcohol and cannabis use in adolescents was assessed using multivariate multilevel regression models.

**Results:**

A total of 7,538 tenth-grade students from 118 schools were included in the study. The 16-item scale of parenting practices showed good internal consistency (Omega total = 0.84), and three factors representing *Relationship between parents and adolescents*, *Norms and monitoring*, and *Parents knowing their children’s friends and the parents of their children’s friends*. High total scores of parenting were associated with lower odds of lifetime alcohol use (OR 0.57; 95% CI: 0.49–0.65), past-month alcohol use (OR 0.63; 95% CI: 0.57–0.70), lifetime drunkenness (OR 0.64; 95% CI: 0.58–0.72), and lifetime cannabis use (OR 0.54; 95% CI: 0.47–0.61). Above median scores on each parenting subscale were associated with significantly lower odds of substance use. The strongest associations were observed for the subscale *Norms and monitoring*. Interactions between parenting and gender showed a significantly stronger effect of parenting practices on alcohol and cannabis use among girls.

**Conclusion:**

Different types of parenting practices were associated with a lower prevalence of adolescent alcohol and cannabis use. Improving parenting practices has the potential to prevent adolescent substance use in Chile, especially among girls.

## 1. Introduction

Substance use often begins during adolescence (Degenhardt et al., 2008; Merikangas et al., 2010). At this age, vulnerability to experimentation, risk of substance use disorders, and sensitivity to neurotoxicity are high (Koob and Volkow, 2016; Volkow et al., 2016; Spear, 2018). Among secondary school students between 8th and 12th grade in Chile, the prevalence of alcohol use was 57% in the past year and 31% in the past month, of which 62% had taken five or more standard drinks (Servicio Nacional para la Prevención y Rehabilitación del Consumo de Drogas y Alcohol, 2018). Cannabis use is also frequent in adolescents and is associated with adverse outcomes (Silins et al., 2014; Volkow et al., 2014; Degenhardt et al., 2016; Hall and Lynskey, 2016). In recent years, the use of cannabis among secondary school students in Chile has increased. It increased from 14.8% in 2001 to 30.9% in 2017 (Servicio Nacional para la Prevención y Rehabilitación del Consumo de Drogas y Alcohol, 2018; Libuy et al., 2020), while the average one-year prevalence of adolescent cannabis use worldwide was 4.7% (United Nations Office on Drugs and Crime, 2020). Initiation of substance use at a young age increases the risk of developing substance use disorders and adverse long-term outcomes (Volkow et al., 2014; Jordan and Andersen, 2017; Levine et al., 2017; Spear, 2018). Among tenth-grade students in Chile, the age of onset of substance use is associated with suicide-related behaviors (Nuñez et al., 2022). Therefore, prevention interventions should prioritize children and adolescents (Degenhardt et al., 2016; Volkow et al., 2016; UNODC and WHO, 2018), and prevention planning for adolescents requires identifying risk and protective factors (Cleveland et al., 2009; Harrop and Catalano, 2016; Chadi et al., 2018).

Family factors are determinants of substance use among children and adolescents. Existing literature distinguishes parenting styles and practices as key determinants of abuse. While parenting styles could range from neglectful to authoritative, parenting practices are actions, behaviors, and rules that parents exert to regulate the personal and social acts of their child. These include parental communication, parental monitoring, and parental knowledge, which delay risk behavior in youth, control the behavior of youth at risk, and promote positive youth development (Ryan et al., 2015). Parental practices are among the key protective factors for adolescent substance use that are modifiable (Harrop and Catalano, 2016; Gaete and Araya, 2017; Chadi et al., 2018). A systematic review of longitudinal studies showed that parental monitoring and involvement, good parent-adolescent relationship, disapproval of adolescent drinking, and parental support and communication were associated with lower levels of alcohol use and delay of alcohol use initiation (Ryan et al., 2010). A more recent meta-analysis concluded that parental monitoring, parent–child relationship quality, parental support, and parental involvement were protective factors for alcohol use among adolescents (Yap et al., 2017). However, evidence from Latin America is still scarce, and family factors may be significant in Latin America. Most of the evidence comes from the Latino population in the United States (Orpinas et al., 2013), and most instruments have not been validated in Latin America. A study from Chile, Mexico, Spain, and Peru showed that parental monitoring and affection were related to constructive leisure time activities and substance use prevention (Belintxon et al., 2020). Authoritative, authoritarian, and indulgent parenting styles had protective effects against multiple drug use compared to neglectful parenting in a study from Brazil (Valente et al., 2017); authoritative parenting was also protective against alcohol use in adolescents from Brazil (Zuquetto et al., 2019). Authoritative style parenting among mothers was associated with stopping or reducing adolescent alcohol use, but the cessation of cocaine or crack required the presence of a strong father figure (Benchaya et al., 2019). In Colombia, adolescents who reported favorable attitudes of parents toward the use of drugs and alcohol had elevated risks of use with an odds ratio of over 3.5 (Montero Zamora et al., 2018). In Argentina, maternal demandingness was associated with lower smoking, drinking, and drug use among eighth-grade students and paternal demandingness with lower binge drinking, but no interactions were found between parenting behavior and students’ gender (Peña et al., 2017). In Mexico, data from fifth- and sixth-grade students showed that lower levels of direct supervision were associated with higher risks of illicit substance use (Vázquez et al., 2019).

Positive outcomes have been reported for a community prevention model of substance use in adolescents based on the assessment of specific risk and protective factors drawn from local surveys among adolescents called Planet Youth, which uses the Islanding Prevention Model (Sigfúsdóttir et al., 2009; Kristjansson et al., 2020a,b). The surveys include a scale assessing perceived parental practices. To our knowledge, the psychometric properties of the parenting scale as part of the questionnaire applied in the Icelandic Prevention Model have not yet been published in the original Icelandic or in the English versions. However, there have been several studies on the association of parenting as a protective factor with substance use outcomes in the original version of the questionnaire (Kristjánsson and Sigfúsdóttir, 2009). In 2018, six municipalities in Chile implemented this prevention model, with promising results regarding municipal prevention capacities (Contreras et al., 2022; Libuy et al., 2023). However, the parenting scale contained in the survey has not yet been validated in a Latin American context. Using the psychometric properties of this parenting scale to assess its association with substance use outcomes can help other countries or communities implement the Icelandic Prevention Model, and it may even facilitate using the perceived parenting scale as a standalone instrument.

The present study aimed to (1) assess the psychometric properties of the parenting scale of the Planet Youth survey and (2) assess the association of different parental practices with alcohol and cannabis use in Chile.

## 2. Methods

### 2.1. Design and sample

We conducted a school-based survey within a prevention-in-action process. A group of researchers developed the survey in Iceland (Sigfúsdóttir et al., 2009; Kristjansson et al., 2020a,b). In 2018, the implementation of this community prevention model of substance use in adolescents was initiated in six municipalities of the Metropolitan Region of Greater Santiago in Chile based on a collaboration between the Icelandic Centre for Social Research and Analysis (ICSRA) and the Universidad de Chile. The prevention action included a survey of tenth-grade students conducted every 2 years to base the community prevention work on local data and evaluate the prevention process.

### 2.2. Survey and procedure

The prevention teams of the municipalities invited schools in their localities to participate. When the schools agreed to participate, written information was sent to the parents. Municipal prevention teams coordinated and supervised implementation of the survey in the schools, with the support and guidance of the Universidad de Chile, following the protocols developed by ICSRA (Kristjansson et al., 2013). We translated the English version of the survey provided by ICSRA into Spanish and adapted the linguistic suitability and content for the context in Chile (Supplementary material 1). This process involved an expert panel and included a semantic validation and assessment of understanding through a pilot study at an adolescent healthcare center. The pencil-and-paper surveys applied on-site were administered in all schools in the same week of June 2018 (Libuy et al., 2023). The surveys were applied during class hours inside the classroom, with the teacher present in the room. The responses were confidential and were not observed by the teacher. The answers were not shared among classmates either. The students had enough time (up to 60 min) to answer the survey. The process of applying the survey, its follow-up, the characteristics of the communities, and the prevention process carried out have also been addressed in previous publications (Contreras et al., 2022; Libuy et al., 2023).

After collecting the completed surveys from the schools, they were scanned and sent to ICSRA, which built the database. We removed duplicates and cases with less than 50% of the responses completed.

### 2.3. Ethical approval

The participation of the municipalities, schools, and students was voluntary, using a passive informed consent procedure for the parents and an assent form for the students. The questionnaire was anonymous, protecting the identity of the students. The anonymized data were managed and stored by the research team. The study was approved by the ethics committee of the Hospital Clínico Universidad de Chile (OAIC 981/18).

### 2.4. Measurements

The survey assessed the prevalence of substance use and several protective and risk factors (see Supplementary material). The protective factors included parental support, monitoring and communication, school wellness, and participation in extracurricular activities such as organized sports activities. Among the risk factors were unsupervised leisure time, lifestyle, and substance use among peers (Kristjansson et al., 2013). Outcome variables were the proportion of adolescents with lifetime alcohol use, past-month alcohol use, lifetime drunkenness, past-month drunkenness, lifetime cannabis use, and cannabis use in life ranging from 1 to 40 times or more (categories: 1 to 2 times, 3 to 5, 6 to 9, 10 to 19, 20 to 39, and 40 or more times in life). The independent variable was the score on the parenting scale. The parenting scale comprised 16 items scored on a Likert-like scale from 1 to 4 by the students. Higher scores indicated higher levels of parental support. For the first 5 items, the initial question was “How easy or hard would it be for you to receive the following from your parents?” and the items were as follows: 1. Caring and warmth, 2. Discussions about personal affairs, 3. Advice about studies, 4. Advice about other issues (projects) of yours, and 5. Assistance with things. For items 1 to 5, a score from 1 to 4 was assigned to answer the alternatives: very difficult, rather difficult, rather easy, or very easy. The introductory question to items 6 to 16 was “How do the following statements apply to you?” and the items were as follows: 6. My parents find it important that I do well in my studies, 7. My parents set definite rules about what I can do at home, 8. My parents set definite rules about what I can do outside the home, 9. My parents set definite rules about when I should be home in the evening, 10. My parents know with whom I am in the evenings, 11. My parents know where I am in the evenings, 12. My parents know my friends, 13. My parents know the parents of my friends, 14. My parents often talk to the parents of my friends, 15. My parents and the parents of my friends sometimes meet to talk to one another, and 16. My parents follow what I do in my recreational time. For items 6 to 16, a score from 1 to 4 was assigned to answer the alternatives: applies very poorly to me, applies rather poorly to me, applies rather well to me, or applies very well to me.

### 2.5. Data analysis

Item analysis of the scale was performed, describing the rate of missing responses, mean scores for each item, standard deviation, skew, item difficulty (rate of ideal response), item discrimination, and Cronbach’s alpha if the item was deleted. Reliability for the scale was analyzed using Cronbach’s alpha and calculating the omega. In the item analysis, total parenting scores were treated as a continuous variable. The Kaiser-Meyer-Olkin factor was calculated, and Bartlett’s K-squared before factor analysis. Exploratory factor analysis was run using polychoric matrices, and the factors were retained with Kaiser’s rule of Eigenvalues over one.

The six municipalities were compared by measuring the invariance using multiple-group confirmatory factor analysis. The changes in model fit indices, such as comparative fit index (CFI) and root-mean-square error of approximation (RMSEA), were assessed for measurement invariance by comparing changes between fit indices at baseline and in equality constraint models (thresholds and thresholds and loadings). For testing loading invariance, a change of ≥ −0.010 in CFI, together with a change of ≥0.015 in RMSEA, indicated non-invariance (Svetina et al., 2020).

Multilevel logistic regression models were performed to analyze the association between the outcomes (lifetime alcohol use, past-month alcohol use, lifetime drunkenness, past-month drunkenness, lifetime cannabis use, and cannabis use in life ranging from 1 to 40 or more times, in six dichotomic categories) and the total parenting score. The outcomes were dichotomic, and the reference category was no substance use. In the multilevel logistic regression, the score of the parenting scale was dichotomized to facilitate the interpretation of the results: high (over the median) and low (equal or under the median). We included the following control variables: gender (categorical dichotomous), age (continuous), living with both parents (categorical nominal), educational level of the parents (categorical nominal), employment status of the parents (categorical nominal), friends using substances (categorical dichotomous), school funding (categorical nominal), and municipality (categorical nominal). Adjusted odds ratios (AOR) were calculated for each outcome. In addition, each subscale of parenting was also dichotomized between high (over the median) and low (equal or under the median), and adjusted odds ratios (AOR) were calculated for each outcome. Multicollinearity between independent variables was assessed with the variance inflation factor (VIF). The VIF values were under five. Thus, collinearity was not present. The linear relationship between independent variables and the logit of the outcome was assessed using scatter plots. In addition, no influential values or outliers were observed in the data.

In the present study, we used a multilevel model to control the cluster effects of the schools and municipalities in which the students were from. The multilevel model allows for analyzing data that is not randomly distributed but grouped in clusters. Intra-class correlations were calculated to estimate the proportion of variance explained at the school and municipality levels. Finally, interactions between gender and parenting were explored using multilevel logistic regression models for lifetime alcohol use, past-month alcohol use, lifetime drunkenness, past-month drunkenness, lifetime cannabis use, and cannabis use in life of 10 or more times. The fitted values of the parenting scores were plotted by gender.

The analyses were performed with the software R version 4.0.1 using the packages psych for psychometrics (Revelle, 2023), items, and factor analyses [fa.poly and omega (Zinbarg et al., 2005) functions of the psych package]. Packages lavaan and semTools for software R were used to measure invariance (Svetina et al., 2020). The packages lme4 (Bates et al., 2015) and insight (Lüdecke et al., 2019) were used for multilevel regressions and intra-class correlation, and sjPlot for plot interactions in models. Raw data are available from the corresponding author upon request.

## 3. Results

### 3.1. Sample description

A total of 7,538 tenth-grade students from 118 schools and six municipalities were included. The response rate was 86.9%. The mean age of the participants was 16.0 (SD = 0.7) years; 48.7% were girls and 51.3% were boys. Table 1 summarizes the description of the sample.

**Table 1:** Substance use prevalence and sample characteristics of 7,538 tenth-grade secondary school students in Greater Santiago.

	Municipalities						Total
	1	2	3	4	5	6	
Total number of participants	1728 (22.9%)	2,438 (32.3%)	1,220 (16.2%)	472 (6.3%)	348 (4.6%)	1,332 (17.7%)	**7,538 (100%)**
Girls	50.6%	49.6%	52.6%	35.9%	44.8%	46.7%	**48.6%**
Mean age (sd)	16.0 (0.7)	16.1 (0.6)	16.1 (0.5)	16.2 (0.9)	16.2 (0.9)	15.9 (0.7)	**16 (0.7)**
Schools	21 (17.8%)	50 (42.4%)	19 (16.1%)	4 (3.4%)	7 (5.9%)	17 (14.4%)	**118 (100%)**
*Funding of schools*							
Private	15	41	19	0	0	15	**90**
Municipal	6	9	0	4	7	2	**28**
Lifetime alcohol use (%)	80.0	84.1	81.4	73.5	66.4	76.5	**79.8**
Past-month alcohol use (%)	42.5	53.3	51.4	38.7	28.8	37.1	**45.5**
Lifetime drunkenness (%)	40.7	47.7	48.6	39.3	32.5	36.0	**42.2**
Past-month drunkenness (%)	15.6	22.0	21.4	16.0	6.8	11.2	**17.2**
Lifetime cannabis use (%)	30.3	25.5	20.3	38.2	30.8	37.3	**27.9**
Cannabis use ten or more times (%)	13.0	10.3	7.2	19.8	14.2	15.3	**11.2**

The total numbers for all six municipalities are given in bold.

### 3.2. Item analysis of the parenting scale

The 16 items of the parenting scale had a mean response rate of 98.5%; on average, 77% of the answers were the expected ideal alternative (score = 4). Table 2 shows response rates, item discrimination, and the expected response rates for each item (also called item difficulty). The mean sum score was 49.1 (SD = 7.5), and the median was 50. The total scale of 16 items showed good internal consistency with a Cronbach’s alpha of 0.84 (95% CI: 0.83–0.85). The alpha, if items were deleted, was ≤0.84 for each item. The omega total was 0.84, and the omega hierarchical was 0.54.

**Table 2:** Item analysis of a parenting scale in 7538 tenth-grade secondary school students in Greater Santiago.

	Item analysis						
	Missing	Mean	SD	Skew	Item difficulty	Item discrimination	α if deleted
Item 1	1.30	3.45	0.74	−1.29	0.86	0.486	0.830
Item 2	1.39	2.83	0.97	−0.37	0.71	0.494	0.829
Item 3	1.43	3.31	0.82	−1.06	0.83	0.461	0.831
Item 4	1.41	3.16	0.9	−0.86	0.79	0.519	0.827
Item 5	1.43	3.29	0.83	−1.02	0.82	0.519	0.827
Item 6	1.21	3.72	0.55	−2.16	0.93	0.257	0.840
Item 7	1.38	3.12	0.91	−0.73	0.78	0.389	0.835
Item 8	1.31	3.11	0.94	−0.77	0.78	0.457	0.831
Item 9	1.53	3.25	0.92	−1.01	0.81	0.408	0.834
Item 10	1.54	3.46	0.83	−1.5	0.87	0.436	0.832
Item 11	1.71	3.56	0.74	−1.73	0.89	0.462	0.831
Item 12	1.64	3.39	0.83	−1.21	0.85	0.480	0.830
Item 13	1.43	2.61	1.02	−0.1	0.65	0.492	0.829
Item 14	1.63	1.98	0.93	0.61	0.49	0.485	0.829
Item 15	1.76	1.69	0.92	1.15	0.42	0.381	0.835
Item 16	1.50	3.10	0.94	−0.74	0.77	0.525	0.827

### 3.3. Factor analysis of the parenting scale

The Kaiser-Meyer-Olkin factor of 0.84 and Bartlett’s K-squared = 4283.3 (df = 15; *p* < 0.001) indicated appropriateness for the factor analysis. Exploratory analysis retained three factors. Factor 1: Items 1 to 5; Factor 2: items 6 to 11 and item 16; and Factor 3: items 12 to 15, with eigenvalues of 1.6, 1.5, and 1.5, respectively. Factor 1 was the *Relationship between parents and adolescents*. Factor 2 reflected parental *Norms and monitoring*. Factor 3 was *Parents knowing their children’s friends and the parents of their children’s friends*. Factor 1 showed high loadings, with unweighted least squares (ULS) over 0.73; Factor 2 had loadings between 0.46 and 0.78; and Factor 3 had loadings between 0.55 and 0.93 (Table 3). RMSEA index was 0.144, and the Tucker-Lewis index of factoring reliability was 0.723. The proportion of the explained variance for each factor was 36% for Factor 1, 34% for Factor 2, and 30% for Factor 3. The internal consistency for each subscale was a total of omega 0.84 for Factor 1, omega 0.77 for Factor 2, and omega 0.81 for Factor 3; and omega hierarchical 0.34 for Factor 1, 0.30 for Factor 2, and 0.26 for Factor 3.

**Table 3:** Standardized loading of factors.

	ULS1	ULS2	ULS3	h2	u2	Complexity
Item 1	0.74	0.01	0.05	0.58	0.42	1.0
Item 2	0.78	−0.04	0.03	0.60	0.40	1.0
Item 3	0.73	0.07	−0.07	0.54	0.46	1.0
Item 4	0.87	−0.02	−0.03	0.72	0.28	1.0
Item 5	0.80	0.00	0.03	0.65	0.35	1.0
Item 6	0.13	0.46	−0.13	0.24	0.76	1.3
Item 7	0.03	0.64	−0.08	0.40	0.60	1.0
Item 8	0.02	0.70	−0.02	0.50	0.50	1.0
Item 9	−0.04	0.78	−0.09	0.56	0.44	1.0
Item 10	−0.01	0.63	0.13	0.46	0.54	1.1
Item 11	−0.01	0.67	0.14	0.52	0.48	1.1
Item 12	0.10	0.19	0.55	0.48	0.52	1.3
Item 13	−0.01	0.05	0.83	0.72	0.28	1.0
Item 14	−0.01	−0.03	0.93	0.84	0.16	1.0
Item 15	0.02	−0.06	0.77	0.57	0.43	1.0
Item 16	0.12	0.50	0.22	0.44	0.56	1.5

ULS, Unweighted Least Squares; h2, communality; u2, specific variance.

Low inter-factor correlations were observed between Factor 1 and Factor 2 (0.39), between Factor 1 and Factor 3 (0.33), and between Factor 2 and Factor 3 (0.35).

The measurement invariance results between municipalities indicated changes under 0.01 from baseline CFI (0.923) compared to thresholds (0.919) and thresholds and loadings (0.922) in equality constraints models; and additionally, RMSEA indicated changes under 0.015 from baseline (0.093) compared to thresholds (0.089), and thresholds and loadings (0.084) in equality constraints models.

### 3.4. Association between parenting and substance use

The total parenting score reported was associated with all outcomes of alcohol use: lifetime alcohol use, past month alcohol use, and lifetime and past month drunkenness. Higher scores of parenting on each subscale were associated with lower probabilities of alcohol use. Table 4 reports the adjusted odds ratios (AORs) for parenting. Among all alcohol use outcomes, lifetime alcohol use had the strongest association. Regarding the different aspects of parenting, the subscale *Norms and monitoring* had the strongest association with alcohol use. The intra-class correlation coefficients observed for the alcohol use outcomes were between 5 and 7% at the school level and lower at the municipality level (see Table 4).

**Table 4:** Association between parenting and alcohol use in 7538 tenth-grade secondary school students in Greater Santiago; multilevel logistic regression analysis.

	Lifetime alcohol		Past-month alcohol		Lifetime drunkenness		Past-month drunkenness	
	AOR	95% CI	AOR	95% CI	AOR	95% CI	AOR	95% CI
Total parenting score*								
Low	1	–	1	–	1	–	1	–
High	0.57	0.49–0.65	0.63	0.57–0.70	0.64	0.58–0.72	0.63	0.55–0.73
Subscale relationship*								
Low	1	–	1	–	1	–	1	–
High	0.81	0.71–0.92	0.85	0.76–0.95	0.81	0.73–0.91	0.80	0.70–0.92
Subscale norms and monitoring*								
Low	1	–	1	–	1	–	1	–
High	0.52	0.45–0.59	0.57	0.51–0.63	0.58	0.52–0.65	0.55	0.48–0.63
Subscale knowledge about friends*								
Low	1	–	1	–	1	–	1	–
High	0.63	0.55–0.72	0.75	0.67–0.84	0.80	0.71–0.89	0.80	0.69–0.92
ICC**								
Schools	6.4%		5.5%		5.3%		6.9%	
Municipalities	2.8%		3.1%		0.7%		3.1%	

*Adjusted for gender, age, living with both parents, parents’ education, employment status of parents, friends using substances, school funding, and municipality.

**Intra-class correlation coefficient for the null model in each outcome.

Higher parenting scores were associated with lower odds of cannabis use. For lifetime cannabis use, the AOR of a high total parenting score was 0.54 (95% CI: 0.47–0.61); and the AOR of the subscale *Relationship between parents and adolescents* was 0.69 (95% CI: 0.70–0.90); the AOR of the subscale *Norms and monitoring* was 0.50 (95% CI: 0.44–0.57); and the subscale *Parents knowing their children’s friends and the parents of their children’s friends* was 0.57 (95% CI: 0.50–0.65). Table 5 presents AORs for the total parenting score and subscales with outcomes representing different frequency categories of lifetime cannabis use. For any lifetime cannabis use, high parenting scores had an AOR similar to the alcohol use outcomes. However, parenting had lower AORs for more frequent lifetime cannabis use (see Table 5). All parenting subscales were significantly associated with cannabis use in any frequency, similar to the total parenting score, except for the subscale *Relationship between parents and adolescents* and cannabis use in life ranging from 1 to 2 times. The subscale *Norms and monitoring* had the strongest association with all categories of cannabis use frequencies (Table 5).

**Table 5:** Association between parenting and cannabis use in 7538 tenth-grade secondary school students in Greater Santiago; multilevel logistic regression analysis.

	1–2 times in life		3–5 times in life		6–9 times in life		10–19 times in life		20–39 times in life		40 or more times	
	AOR	95% CI	AOR	95% CI	AOR	95% CI	AOR	95% CI	AOR	95% CI	AOR	95% CI
Total parenting score*												
Low	1	–	1	–	1	–	1	–	1	–	1	–
High	0.70	0.58–0.84	0.68	0.53–0.87	0.54	0.40–0.73	0.32	0.23–0.45	0.41	0.28–0.58	0.36	0.27–0.48
Subscale relationship*												
Low	1	–	1	–	1	–	1	–	1	–	1	–
High	0.84	0.70–1.00	0.68	0.53–0.87	0.62	0.45–0.84	0.62	0.46–0.84	0.51	0.36–0.73	0.57	0.44–0.75
Subscale norms and monitoring*												
Low	1	–	1	–	1	–	1	–	1	–	1	–
High	0.61	0.51–0.72	0.63	0.49–0.79	0.56	0.42–0.75	0.36	0.27–0.49	0.40	0.28–0.57	0.31	0.23–0.41
Subscale knowledge about friends*												
Low	1	–	1	–	1	–	1	–	1	–	1	–
High	0.73	0.60–0.88	0.69	0.53–0.89	0.47	0.33–0.66	0.43	0.31–0.61	0.37	0.25–0.56	0.45	0.33–0.61
ICC**												
Schools	5.2%		10.7%		13.0%		15.2%		21.0%		34.8%	
Municipalities	0.7%		0.7%		0.0%		0.7%		0.0%		3.5%	

*Adjusted for gender, age, living with both parents, parents’ education, employment status of parents, friends using substances, school funding, and municipality.

**Intra-class correlation coefficient for the null model in each outcome.

Intra-class correlation coefficients observed in lifetime cannabis use were 15.8% at the school level and 1.4% at the municipality level. We observed higher intra-class correlation coefficients at the school level in the categories indicating more frequent lifetime cannabis use (Table 5). The intra-class coefficients at the municipality level were lower than those at the school level for the cannabis use outcomes. The school level explained more variance of cannabis use than alcohol use.

### 3.5. Gender interactions

**Figure 1:**
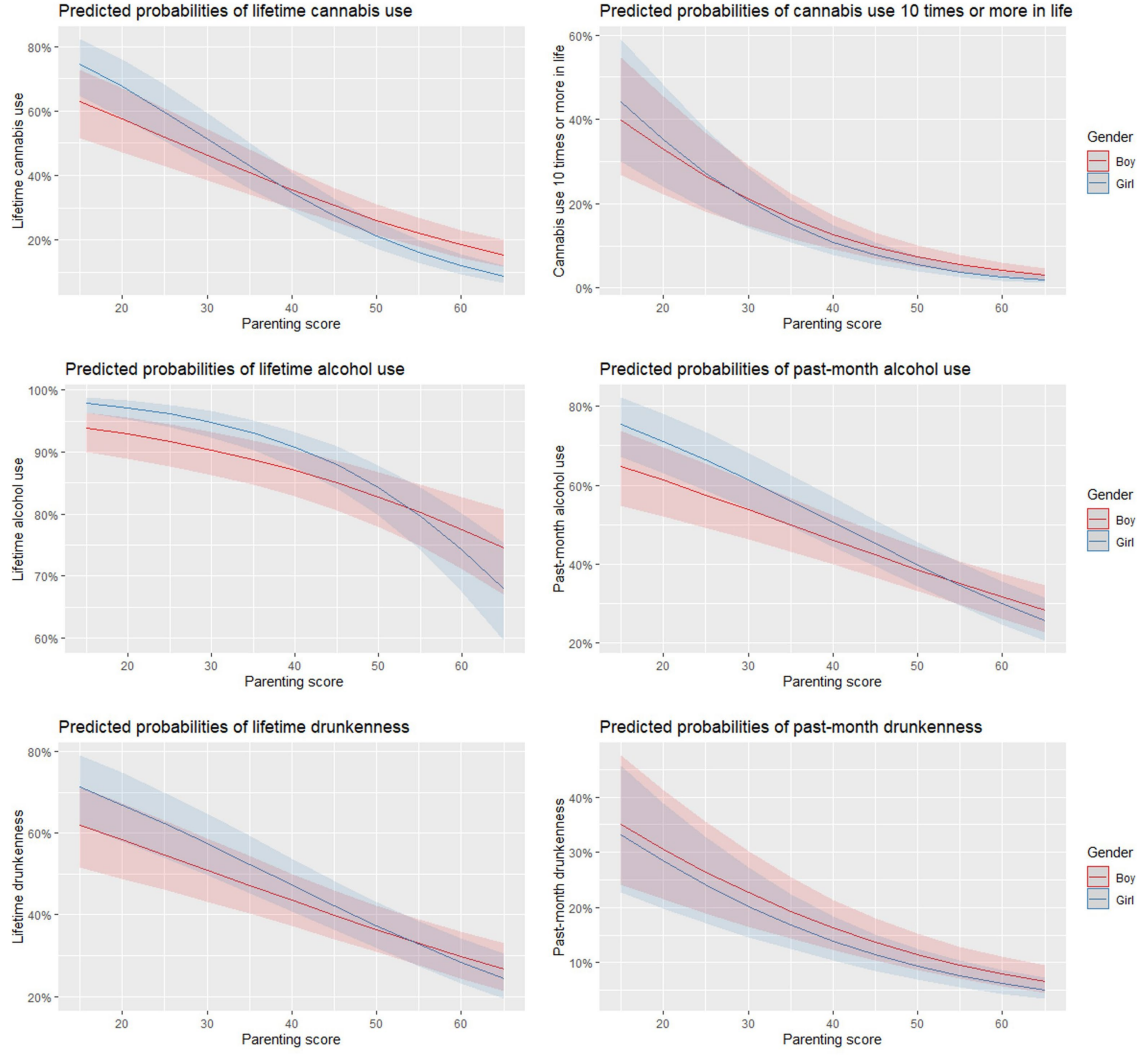
This figure shows the interaction between parenting scores and gender for different substsance use outcomes using multilevel regression models. Significantly stronger effects of parenting in girls is observed for lifetime (*p* = 0.01) and past month alcohol use (*p* = 0.005), lifetime cannabis use (*p* = 0.005), and cannabis use 10 times or more in life (*p* = 0.021).

The association of high parenting scores with less adolescent alcohol and cannabis use was significantly stronger for girls than boys. In multilevel regression models, significant interactions between parenting and gender were observed, with a greater effect of parenting on substance use in girls than in boys for lifetime alcohol use (*p* = 0.01), past-month alcohol use (*p* = 0.005), lifetime cannabis use (p = 0.005), and cannabis use in life of more than 10 times (*p* = 0.021). However, the interaction was not significant for lifetime drunkenness (*p* = 0.235) and drunkenness in the past month (*p* = 0.224). Figure 1 shows the interactions between parenting and gender for substance use outcomes.

## 4. Discussion

### 4.1. Main findings

This study presents the psychometric properties of a 16-item scale to assess perceived parental practices in tenth-grade secondary school students in Chile. The scale is part of a larger questionnaire on risk and protective factors of adolescent substance use. It showed good internal consistency and a three-factor structure in Chile: 1. *Relationship between parents and adolescents*; 2. *Norms and monitoring*; and 3. *Parents knowing their children’s friends and the parents of their children’s friends*. Parental practices were associated with alcohol and cannabis use among adolescents. The items related to *Norms and monitoring* had the strongest association with substance use. Parental practices showed a stronger association with substance use in girls than in boys.

### 4.2. Comparison with the literature

Few parenting instruments have strong psychometric properties, and very few have been tested in the Spanish language. In Mexican-American populations, cross-language measurement equivalence was investigated for warmth, consistent discipline, harsh parenting, and parental monitoring, showing reliability between Cronbach’s alpha 0.7 to 0.8 for each scale and adequate convergent construct validity equivalence across languages (Nair et al., 2009). The Parenting Children and Adolescents (PARCA) scale was nearly invariant in the Latino population in the United States in the Spanish version, showing a reliability of Cronbach’s alpha 0.95, and the authors described a confirmatory factor analysis with good model fit for a three-factor structure: supporting good behavior, proactive parenting, and setting limits (Ringle et al., 2019). Outside the US, perceived parental monitoring was assessed in Mexico City with a scale adapted from Kerr and Stattin that showed good reliability with a Cronbach’s alpha of 0.82 (Stattin and Kerr, 2000; Kerr et al., 2010), similar to the reliability of the scale used in the present study. In Colombia, however, based on the Communities That Care survey, the measure of attitudes of parents towards adolescent drug use had limited reliability with a Cronbach’s alpha coefficient of 0.57 (Mejía-Trujillo et al., 2015).

Parenting scores were associated with all substance use outcomes in our study, indicating construct validity. The odds ratios reported in this study had small to medium effect sizes (Chen et al., 2010). A meta-analysis of longitudinal studies on alcohol use in adolescents reported a small pooled effect size for the predictors: parent–child relationship quality, parental involvement, parental monitoring, and parental support, with correlation coefficients between −0.1 and − 0.2 (Yap et al., 2017). In line with our results, a higher effect size was reported for parental monitoring. Regarding cannabis, it has been reported that general support from parents and parents knowing their children’s friends were preventive for lifetime use (OR 0.72 and 0.27, respectively) and for past month use (OR 0.68 and 0.21, respectively) (Vermeulen-Smit et al., 2015). Cannabis-specific rules were preventive for cannabis use in the past month with an OR of 0.6. International literature is consistent with our findings about larger effect sizes of parental practices on cannabis compared to alcohol use.

Interestingly, the present study showed that the association of parenting with cannabis use was stronger than the association of parenting with alcohol use; and more frequent cannabis use was more strongly associated with parenting, suggesting a dose-dependent relationship. A high score on the parenting scale was more protective for more severe substance use. Differential effects of parenting on alcohol and cannabis use in adolescents could be related to the legal status of the substances. The use of cannabis in adolescents may be considered more problematic than the use of alcohol. Although legalization is debated and its use decriminalized, cannabis is still an illegal drug for recreational use in Chile and is less normalized than alcohol (Mundt et al., 2023).

The cluster effect of the schools on substance use, which may also reflect the effect of peers, was higher for cannabis than for alcohol use and especially high for frequent cannabis use. Previous research from Chile also found strong effects of the school environment on cannabis use, compared to alcohol use (Gaete and Araya, 2017; Libuy et al., 2020). The use of cannabis among friends was identified as the most important risk factor for adolescent substance use in Chile (Libuy et al., 2020). However, in line with the theory of normalization, a higher prevalence of cannabis use showed a reduction in the association between cannabis use and cannabis use among friends (Sznitman et al., 2013). The impact of peers on alcohol use in adolescents was lower than on cannabis use (Mason et al., 2017). Parental practices may moderate the effect friends may have on substance use in adolescents (Kiesner et al., 2010; Boyd-Ball et al., 2014).

Our study showed that gender significantly interacted with the association of parenting and substance use of adolescents in Chile; higher parenting scores were more protective for girls than boys regarding alcohol and cannabis use. The male gender role has been linked to more alcohol use and stronger expression of the female role to less alcohol use (Seedat et al., 2009; Shakya et al., 2019). Lower perceived parental monitoring and weaker perceived family relationships were more strongly associated with drinking profiles among females than among males (Strunin et al., 2015). The effect of parental monitoring on criminal conduct was also stronger for girls than for boys (Kerr et al., 2010). Parents appeared to protect girls more than boys. Connectedness to parents and parental control also had a positive effect on risk behaviors in girls. Adolescent disclosure to parents had a stronger association with criminal conduct in girls than in boys (Kapetanovic et al., 2019). Further, in Iceland, girls received more support and monitoring from their parents compared to boys (Kristjánsson and Sigfúsdóttir, 2009).

### 4.3. Strengths and limitations

This is the first validation of the parenting scale administered as part of Planet Youth’s Icelandic Prevention Model in Spanish and Latin America. We used a large sample of adolescents from many schools and diverse municipalities in Chile.

The cross-sectional study design has limitations regarding the inference of causality. The reverse causality or bidirectionality of influences is also possible. Further limitations arise from the self-report survey design without direct assessment of the parents. The adolescents perceived the parenting practice. We did not assess careless or insufficient effort responding, which may affect the study’s internal validity and lead to a response bias. In future studies, cleaning the data with a careless response analysis could help to detect bias, ensure greater internal validity, and determine whether realistic and accurate data are available (Merino-Soto et al., 2021; Alarcon and Lee, 2022). Lastly, only six municipalities were studied. Therefore, the estimate of the effect at the municipal level may not be accurate.

## 5. Conclusion

The Spanish version of the parenting scale used as part of the Icelandic Prevention Model of adolescent substance use is reliable and a valid instrument to assess parenting in Latin America. Our study highlights the role of the parent-adolescent relationship, parental norms and monitoring, and parents knowing their children’s friends in alcohol and cannabis use prevention among adolescents. The identification of these local protective factors can guide future prevention work with families in Chile and other Latin American contexts.

Our results show the potential of interventions aimed at improving parental practices for substance use prevention in adolescents. Future research should address strategies to improve parenting practices. Collective agreements among parents of adolescents could be a way forward to make parenting more effective.

## Data availability statement

The raw data supporting the conclusions of this article will be made available by the authors, without undue reservation.

## Ethics statement

The studies involving humans were approved by Comité de ética, Hospital Clínico Universidad de Chile. The studies were conducted in accordance with the local legislation and institutional requirements. The Ethics Committee/Institutional review board waived the requirement of written informed consent for participation from the participants or the participants’ legal guardians/next of kin because passive informed consent was given by the parents, informed assent was obtained by the adolescent study participants.

## Author contributions

NL, VG, AM, CI, and JG: conceptualization and designed the research. NL, VG, AA, CI, LC, PD, and AM: methodology and data collection. NL, AM, and JG contributed to the data analysis. NL, VG, AM, and JG wrote the manuscript. All authors contributed to the article revision and approved the submitted version.

## Funding

The research was funded by the Agencia Nacional de Investigación y Desarrollo, Chile, grant scheme FONIS, grant number SA19I0152. NL received funding from the Agencia Nacional de Investigación y Desarrollo, Chile, CONICYT PFCHA/DOCTORADO NACIONAL/2018–21180520. JG was funded by ANID – Millennium Science Initiative Program – NCS2021_081.

## Conflict of interest

The authors declare that the research was conducted in the absence of any commercial or financial relationships that could be construed as a potential conflict of interest.

## Publisher’s note

All claims expressed in this article are solely those of the authors and do not necessarily represent those of their affiliated organizations, or those of the publisher, the editors and the reviewers. Any product that may be evaluated in this article, or claim that may be made by its manufacturer, is not guaranteed or endorsed by the publisher.

## Supplementary material

Supplementary Data Sheet 1 Survey Planet Youth, Universidad de Chile 2018.
