# Supplementary material for: Parental practices and their association with alcohol and cannabis use among adolescents in Chile
Source: Front Psychol. 2023 Sep 11;14:1209584. doi: 10.3389/fpsyg.2023.1209584 (PMC10520567; doi:10.3389/fpsyg.2023.1209584)
Supplement: Supplementary Data Sheet 1 — Survey Planet Youth, Universidad de Chile 2018. [file Data_Sheet_1.pdf]

PLANET Youth
EVIDENCE BASED DRUG PREVENTION

UNIVERSIDAD DE CHILE

Planet Youth 2018

Juventud y bienestar

Una encuesta sobre la vida y las condiciones de vida de los jóvenes

A los estudiantes

Este cuadernillo contiene varias preguntas para responder. Estas preguntas son sobre tus opiniones en varios temas, así como sobre diferentes tipos de actividades que realizas o en las que podrías estar involucrado. Probablemente nunca has participado de una encuesta similar, pero esperamos que puedas responder muy a conciencia porque tus respuestas son muy importantes. Es importante que respondas las preguntas de la forma en que mejor describa tu opinión. Esto es completamente diferente a una prueba, ***no hay respuestas más correctas que otras***. Lo único importante aquí es conocer tus opiniones.

La mayoría de las preguntas tienen varias opciones para que puedas elegir tu respuesta, pero debes elegir solo una de ellas. Pon una **X** en el casillero que está al lado de la respuesta que has escogido. No uses un lápiz muy suave, y no rellenes completamente el casillero que has elegido. Si cambias de opinión, debes borrar la respuesta o rellenar completamente el casillero con la respuesta equivocada. Unas pocas preguntas no son de alternativas, y tu debes escribir la respuesta. En tales casos, debes escribir claramente en letras mayúsculas poniendo solo una letra por casillero. Te pedimos que respondas todas las preguntas lo mejor que puedas. Si sientes que ninguna de las alternativas describe tu opinión precisamente, elige la respuesta que tu pienses que está más cercana a tu opinión.

Va a ser imposible asociar tus respuestas contigo, en otras palabras, nadie que tu conozcas, ni tus profesores, padres, familiares o amigos, podrán acceder a tus respuestas personales. Asegúrate de ***no escribir tu nombre ni ninguna identificación personal*** en las hojas del cuestionario ni en los sobres que te entregaron. Cuando hayas terminado de responder todas las preguntas, pon el cuestionario en el sobre, séllalo completamente y déjalo en tu mesa. Los sobres serán recogidos cuando todos hayan terminado.

Si tienes alguna duda sobre alguna de las preguntas, cierra tu cuadernillo y levanta la mano. Un encargado se acercará a ti con un cuadernillo para ayudarte sin ver tus respuestas.

Con nuestros más cordiales saludos,

Equipo Planet Youth Chile

POR FAVOR ANTES DE CONTESTAR LAS PREGUNTAS, LEE CUIDADOSAMENTE LAS INSTRUCCIONES DE LA PÁGINA ANTERIOR.
Contesta cada pregunta marcando con una X en el recuadro respectivo

1. **¿Eres hombre o mujer?**
   ☐ Hombre ☐ Mujer

2. **¿Cuál es tu año de nacimiento?** (Elige solo UNA opción)
   ☐ 2000 ☐ 2001 ☐ 2002 ☐ 2003 ☐ 2004 ☐ 2005

3. **¿En qué curso estás?** (Elige solo UNA opción)
   ☐ 7º Básico ☐ 8º Básico ☐ 1º Medio ☐ 2º Medio ☐ 3° Medio

4. **Vives con...** (Elige solo UNA opción, la mejor respuesta)
   - ☐ Con madre y padre
   - ☐ Con madre, sin padre
   - ☐ Con padre, sin madre
   - ☐ Con madre y su pareja
   - ☐ Con padre y su pareja
   - ☐ Con abuelos y padres
   - ☐ Solo con abuelos, sin padres
   - ☐ Con amigos
   - ☐ Vivo solo
   - ☐ Vivo de otra forma (familia de acogida, hogar de menores, etc.)

5. **¿Cuál es el mayor nivel de estudios que completó tu madre o apoderada?** (Si fuiste criado por una madre adoptiva, responde por ella. Elige solo UNA opción)
   - ☐ No lo sé / No aplica
   - ☐ Completó estudios en la universidad
   - ☐ Comenzó la universidad pero no terminó
   - ☐ Completó estudios en una carrera técnica
   - ☐ Comenzó una carrera técnica pero no la terminó
   - ☐ Terminó el colegio
   - ☐ No terminó la educación media
   - ☐ Educación básica completa o menos

6. **¿Cuál es el mayor nivel de estudios que completó tu padre o apoderado?** (Si fuiste criado por un padre adoptivo, responde por él. Elige solo UNA opción)

- ☐ No lo sé / no aplica
- ☐ Completó estudios en la universidad
- ☐ Comenzó la universidad pero no terminó
- ☐ Completó estudios en una carrera técnica
- ☐ Comenzó una carrera técnica pero no la terminó
- ☐ Terminó en colegio
- ☐ No terminó la educación media
- ☐ Educación básica completa o menos

7. **¿Cuál es la principal actividad de tu madre o apoderada?** (Elige solo UNA opción, la que se ajuste mejor)

- ☐ Mi Madre trabaja en casa (dueña de casa)
- ☐ Mi Madre trabaja media jornada
- ☐ Mi Madre trabaja jornada completa
- ☐ Mi Madre trabaja en el extranjero
- ☐ Mi Madre no tiene trabajo
- ☐ Mi Madre está discapacitada, no trabaja
- ☐ Mi Madre es estudiante
- ☐ Mi Madre está estudiando y trabajando
- ☐ No lo sé /No aplica

8. **¿Cuál es la principal actividad de tu padre o apoderado?** (elige solo UNA opción, la que se ajuste mejor)

- ☐ Mi Padre trabaja en casa (dueño de casa)
- ☐ Mi Padre trabaja media jornada
- ☐ Mi Padre trabaja jornada completa
- ☐ Mi Padre trabaja en el extranjero
- ☐ Mi Padre no tiene trabajo
- ☐ Mi Padre esta discapacitado, no trabaja
- ☐ Mi Padre es estudiante
- ☐ Mi Padre está estudiando y trabajando
- ☐ No lo sé / No aplica

9. **¿Dónde naciste?** (Elige UNA opción y escribe la respuesta si corresponde)
- ☐ En este país
- ☐ En otro país ☐☐☐☐☐☐☐☐☐☐☐☐☐☐☐☐☐

10. **¿Tus padres nacieron y crecieron en este país?** (Elige UNA opción en CADA categoría)

	Sí	No
a) Mi madre nació en este país	☐	☐
b) Mi madre creció en este país	☐	☐
c) Mi padre nació en este país	☐	☐
d) Mi padre creció en este país	☐	☐

11. **¿Se habla español en tu casa?** (Elige solo UNA opción)
- ☐ Sí, solo español
- ☐ Sí, junto con otro idioma
- ☐ No, solo se habla otro idioma

12. **¿Cómo crees tú que está económicamente tu familia en comparación a otras familias en el país?** (Elige solo UNA opción)
- ☐ Mucho mejor
- ☐ Bastante mejor
- ☐ Un poco mejor
- ☐ Similar a otros
- ☐ Un poco peor
- ☐ Bastante peor
- ☐ Mucho peor

13. **¿A qué grupo o comunidad religiosa perteneces?** (Elige solo UNA opción)
- ☐ Católica
- ☐ Evangélica o Protestante
- ☐ Testigo de Jehová
- ☐ Ortodoxa
- ☐ Mormona
- ☐ Judia
- ☐ Otra
- ☐ No pertenezco a una comunidad religiosa

14. ¿Vas a un colegio de tu barrio? (Elige solo UNA opción)

☐ Sí ☐ No

15. ¿Qué tan buen alumno crees que eres, comparado con otras personas de tu edad? (Elige solo UNA opción)

Excelente, probablemente soy uno de los mejores	Muy por sobre el promedio	Sobre el promedio	En el promedio	Bajo el promedio	Muy por debajo del promedio	Malo, probablemente soy uno de los peores
☐	☐	☐	☐	☐	☐	☐

16. ¿Cuántos días completos has estado ausente del colegio durante los últimos 30 días? (Elige UNA opción en CADA categoría)

	Ninguno	1 día	2 días	3-4 días	5 o más días
a) Por enfermedad	☐	☐	☐	☐	☐
b) Por hacer la cimarra	☐	☐	☐	☐	☐
c) Por otras razones	☐	☐	☐	☐	☐

17. ¿Qué tan bien te representan las siguientes frases? (Elige UNA opción en CADA categoría)

	Me representa casi siempre	Me representa frecuentemente	Me representa algunas veces	Me representa pocas veces	Casi nunca me representa
a) Creo que estudiar en el colegio no tiene sentido	☐	☐	☐	☐	☐
b) Estoy aburrido(a) de los estudios	☐	☐	☐	☐	☐
c) Estoy mal preparado(a) para las clases	☐	☐	☐	☐	☐
d) Siento que no pongo suficiente esfuerzo en los estudios	☐	☐	☐	☐	☐
e) Encuentro muy fácil los estudios	☐	☐	☐	☐	☐
f) Encuentro muy difícil los estudios	☐	☐	☐	☐	☐
g) Me siento mal en el colegio	☐	☐	☐	☐	☐
h) Quiero abandonar los estudios	☐	☐	☐	☐	☐
i) Quiero cambiarme de colegio	☐	☐	☐	☐	☐
j) Me llevo mal con los profesores	☐	☐	☐	☐	☐

PLANET Youth
EVIDENCE BASED DRUG PREVENTION

UNIVERSIDAD DE CHILE

18. Durante los últimos 7 días, ¿cuántas veces hiciste alguna de las siguientes cosas? (Elige UNA opción en CADA categoría)

	Nunca	Una vez	Dos veces	3 veces	4 veces	5 veces	6 veces	7 veces
a) Estar en casa toda la noche	☐	☐	☐	☐	☐	☐	☐	☐
b) Estar fuera de casa después de las diez de la noche	☐	☐	☐	☐	☐	☐	☐	☐
c) Salir y volver después de media noche	☐	☐	☐	☐	☐	☐	☐	☐

19. ¿Cuán fácil o difícil sería para ti recibir u obtener lo siguiente de tus padres o apoderados? (Elige UNA opción en CADA categoría)

	Muy difícil	Difícil	Fácil	Muy fácil
a) Cariño y calidez	☐	☐	☐	☐
b) Una conversación sobre temas personales	☐	☐	☐	☐
c) Consejos sobre los estudios	☐	☐	☐	☐
d) Consejos sobre otros asuntos (proyectos) tuyos	☐	☐	☐	☐
e) Ayuda con cosas	☐	☐	☐	☐

20. ¿Qué tan fácil o difícil sería para ti recibir u obtener lo siguiente de tus amigos? (Elige UNA opción en CADA categoría)

	Muy difícil	Bastante difícil	Bastante fácil	Muy fácil
a) Cariño y calidez	☐	☐	☐	☐
b) Una conversación sobre temas personales	☐	☐	☐	☐
c) Consejos sobre los estudios	☐	☐	☐	☐
d) Consejos sobre otros asuntos (proyectos) tuyos	☐	☐	☐	☐
e) Ayuda con cosas	☐	☐	☐	☐

21. ¿Cómo se aplican a ti las siguientes frases? (Elige UNA opción en CADA categoría)

	Casi nunca	Pocas veces	Algunas veces	Frecuente-mente	Casi siempre
a) Paso tiempo con mis padres o apoderados en la semana, despues del colegio	☐	☐	☐	☐	☐
b) Paso tiempo con mis padres o apoderados los fines de semana	☐	☐	☐	☐	☐

22. ¿Tus padres o apoderados saben donde estás los sábados en la noche? (Elige solo UNA opción)

Casi nunca	Pocas veces	Algunas veces	Frecuentemente	Casi siempre
☐	☐	☐	☐	☐

23. ¿Cuánto te representa la siguiente frase? "Me siento seguro(a)..." (Elige UNA opción para CADA subcategoría)

	Casi nunca	Pocas veces	Algunas veces	Frecuentemente	Casi siempre
a) en casa	☐	☐	☐	☐	☐
b) en el colegio	☐	☐	☐	☐	☐
c) en mi barrio	☐	☐	☐	☐	☐

25. ¿Cuánto te representan las siguientes frases? (Elige UNA opción en CADA categoría)

	Me representa muy bien	Me representa bien	Me representa poco	Me representa muy poco
a) Mis padres o apoderados encuentran importante que me vaya bien en los estudios	☐	☐	☐	☐
b) Mis padres o apoderados tienen reglas claras sobre lo que puedo hacer en casa	☐	☐	☐	☐
c) Mis padres o apoderados tienen reglas claras sobre lo que puedo hacer fuera de casa	☐	☐	☐	☐
d) Mis padres o apoderados tienen reglas claras sobre cuándo debo estar en casa en la noche	☐	☐	☐	☐
e) Mis padres o apoderados saben con quién estoy cuando salgo de noche	☐	☐	☐	☐
f) Mis padres o apoderados saben dónde estoy cuando salgo de noche	☐	☐	☐	☐
g) Mis padres o apoderados conocen a mis amigos(as)	☐	☐	☐	☐
h) Mis padres o apoderados conocen a los padres de mis amigos(as)	☐	☐	☐	☐
i) Mis padres o apoderados hablan frecuentemente con los padres de mis amigos(as)	☐	☐	☐	☐
j) Mis padres o apoderados y los padres de mis amigos(as) algunas veces se reúnen a hablar entre ellos	☐	☐	☐	☐
k) Mis padres o apoderados están al tanto de lo que hago cuando no estoy en el colegio	☐	☐	☐	☐

25. ¿Cuánto te representan las siguientes frases? (Elige UNA opción en CADA categoría)

	Casi siempre	Frecuente-mente	Algunas veces	Pocas veces	Casi nunca
a) Mis padres o apoderados tienen amigos que viven cerca de nuestra casa	☐	☐	☐	☐	☐
b) Mis padres o apoderados saben el nombre de muchos de nuestros vecinos	☐	☐	☐	☐	☐
c) Mis padres o apoderados algunas veces visitan a algunos de nuestros vecinos	☐	☐	☐	☐	☐
d) Mis vecinos algunas veces visitan a mis padres	☐	☐	☐	☐	☐
e) Algunas veces pedimos prestado cosas a nuestros vecinos (ej. leche o herramientas)	☐	☐	☐	☐	☐
f) Algunas veces nuestros vecinos nos piden cosas prestadas (ej. leche o herramientas)	☐	☐	☐	☐	☐

26. ¿Cuán probable o improbable es que tus vecinos hagan algo si...? (Elige UNA opción en CADA categoría)

	Muy probable	Bastante probable	Ni lo uno ni lo otro	Bastante improbable	Muy improbable
a) ...los jóvenes en el barrio faltan al colegio y se quedan por ahí	☐	☐	☐	☐	☐
b) ...los jóvenes están haciendo grafitis en las casas del sector	☐	☐	☐	☐	☐
c) ...los jóvenes son irrespetuosos con los adultos	☐	☐	☐	☐	☐
d) ...se genera una pelea fuera de tu casa	☐	☐	☐	☐	☐
e) ...alguien está entrando a robar un auto o casa en tu call	☐	☐	☐	☐	☐

27. Por favor responde si lo siguiente te representa y en qué medida. (Elige UNA opción en CADA categoría)

	Casi nunca	Pocas veces	Algunas veces	Frecuente-mente	Casi siempre
a) Mis padres o apoderados están mal económicamente	☐	☐	☐	☐	☐
b) A mis padres o apoderados no les alcanza para tener un auto	☐	☐	☐	☐	☐
c) A mis padres o apoderados les alcanza justo para pagar nuestras necesidades basicas como familia (e.j. comida, vivienda, teléfono)	☐	☐	☐	☐	☐
d) A mis padres o apoderados no les alcanza para pagar por actividades extracurriculares en las que me gustaria participar (e.j. practicar un instrumento musical o deportes)	☐	☐	☐	☐	☐

28. ¿Cuánto te representan las siguientes frases? (Elige UNA opción en CADA categoría)

	Totalmente en desacuerdo	Algo en desacuerdo	Algo de acuerdo	Totalmente de acuerdo
a) Algunas veces es necesario fumar cigarros para no ser excluido en el grupo de compañeros	☐	☐	☐	☐
b) Algunas veces es necesario beber alcohol para no ser excluido en el grupo de compañeros	☐	☐	☐	☐
c) Algunas veces es necesario fumar marihuana para no ser excluido en el grupo de compañeros	☐	☐	☐	☐
d) Algunas veces es necesario hacer la cimarra para no ser excluido en el grupo de compañeros	☐	☐	☐	☐

29. ¿Cuántas horas en promedio duermes cada noche? (Elige solo UNA categoría)

Más de 9 horas	Cerca de 9 horas	Cerca de 8 horas	Cerca de 7 horas	Cerca de 6 horas	Menos de 6 horas
☐	☐	☐	☐	☐	☐

30. Considerando la semana pasada, ¿qué tan bien describen tu estado de ánimo las siguientes frases? (Elige UNA opción en CADA categoría)

	Nunca o Casi nunca	Pocas veces	Algunas veces	Frecuente-mente
a) Me enojaba o irritaba con facilidad	☐	☐	☐	☐
b) Experimenté arrebatos de ira que no podía controlar	☐	☐	☐	☐
c) Quería romper o dañar cosas	☐	☐	☐	☐
d) Tuve una discusión con alguien	☐	☐	☐	☐
e) Le grité a alguien o tiré cosas	☐	☐	☐	☐

31. ¿Qué tan bien te representan las siguientes frases? (Elige UNA opción en CADA categoría)

	Para nada	Bastante mal	Bastante bien	Muy bien
a) Cuando pienso en como me veré en el futuro, me siento satisfecho(a)	☐	☐	☐	☐
b) Muy frecuentemente pienso que soy feo(a) y poco atractivo(a)	☐	☐	☐	☐
c) Estoy feliz con mi cuerpo	☐	☐	☐	☐
d) Estoy feliz con los cambios físicos que han ocurrido en mi cuerpo los últimos años	☐	☐	☐	☐
e) Me siento físicamente fuerte y saludable	☐	☐	☐	☐
f) Estoy contento(a) con mi vida	☐	☐	☐	☐
g) Estoy feliz	☐	☐	☐	☐

32. Considerando las últimas 2 semanas, ¿Con qué frecuencia te ha pasado lo siguiente?

	Nunca	Rara vez	Algunas veces	Frecuente-mente	Siempre
a) Me he sentido optimista sobre el futuro	☐	☐	☐	☐	☐
b) Me he sentido útil	☐	☐	☐	☐	☐
c) Me he sentido relajado(a)	☐	☐	☐	☐	☐
d) He estado afrontando bien mis problemas	☐	☐	☐	☐	☐
e) He estado pensando claro	☐	☐	☐	☐	☐
f) Me he sentido cercano(a) a otras personas	☐	☐	☐	☐	☐
g) He podido tomar decisiones respecto algunas cosas	☐	☐	☐	☐	☐

33. ¿Como calificarías tu salud física? (Elige solo UNA opción)

Muy buena	Buena	Regular	Mala	Muy mala
☐	☐	☐	☐	☐

34. ¿Como calificarías tu salud mental? (Elige solo UNA opción)

Muy buena	Buena	Regular	Mala	Muy mala
☐	☐	☐	☐	☐

35. ¿Cuán de acuerdo o en desacuerdo estas con las siguientes frases? (Elige UNA opción en CADA categoría)

	Totalmente en desacuerdo	Algo en desacuerdo	Neutral	Algo de acuerdo	Totalmente de acuerdo
a) Uno puede romper la mayoría de las reglas si el resto no las sigue	☐	☐	☐	☐	☐
b) Sigo las reglas que quiero seguir	☐	☐	☐	☐	☐
c) Hay muy pocas reglas absolutas en la vida	☐	☐	☐	☐	☐
d) Es difícil confiar en algo, porque todo cambia	☐	☐	☐	☐	☐
e) Nadie sabe que se espera de uno en la vida	☐	☐	☐	☐	☐
f) Uno nunca puede estar seguro de nada en la vida	☐	☐	☐	☐	☐
g) A veces uno necesita romper las reglas para tener éxito	☐	☐	☐	☐	☐
h) Seguir las reglas no asegura éxito	☐	☐	☐	☐	☐

36. ¿Con qué frecuencia sentiste alguna de las siguientes molestias físicas o psicológicas en la semana pasada? (Elige UNA opción en CADA categoría)

	Nunca o Casi nunca	Pocas veces	Algunas veces	Frecuente-mente
a) Me sentí nervioso(a)	☐	☐	☐	☐
b) Sentí miedo repentino sin razón aparente	☐	☐	☐	☐
c) Me sentí tenso(a)	☐	☐	☐	☐
d) Me sentí poco interesado(a) en hacer cosas	☐	☐	☐	☐
e) Tenía poco apetito	☐	☐	☐	☐
f) Me sentí sólo(a)	☐	☐	☐	☐
g) Lloraba fácilmente o quería llorar	☐	☐	☐	☐
h) Tuve problemas para dormir	☐	☐	☐	☐
i) Me sentí triste o decaído(a)	☐	☐	☐	☐
j) No disfrutaba al hacer las cosas	☐	☐	☐	☐
k) Estaba lento(a) o tenía poca energía	☐	☐	☐	☐
l) El futuro parecía sin esperanza	☐	☐	☐	☐
m) Pensé en suicidarme	☐	☐	☐	☐

37. ¿Alguno de los siguientes enunciados se aplica a ti? (Elige UNA opción en CADA categoría)

		Si	No
a)	¿Alguien te ha dicho que está pensando en suicidarse?	☐	☐
b)	¿Alguien de tus conocidos, no cercanos, ha intentado suicidarse alguna vez?	☐	☐
c)	¿Alguien de tus conocidos, no cercanos, se ha suicidado?	☐	☐
d)	¿Alguno de tus amigos o alguien cercano ha intentado suicidarse?	☐	☐
e)	¿Alguno de tus amigos o alguien cercano se ha suicidado?	☐	☐
f)	¿Has pensado alguna vez en suicidarte?	☐	☐
g)	¿Has considerado seriamente suicidarte alguna vez ?	☐	☐
h)	¿Le has dicho alguna vez a alguien que estabas pensando en suicidarte?	☐	☐
i)	¿Has hecho alguna vez un intento de suicidio?	☐	☐
j)	¿Has hecho un intento de suicidio en los últimos 6 meses?	☐	☐

38. Alguna vez en la vida has pensado en hacerte daño intencionalmente (como rasguñarte, quemarte, cortarte, pegarte)

Nunca	1 vez en la vida	2 veces en la vida	3-4 veces en la vida	5 veces o más
☐	☐	☐	☐	☐

39. Alguna vez en la vida te has hecho daño intencionalmente (como rasguñarte, quemarte, cortarte, pegarte)

Nunca	1 vez en la vida	2 veces en la vida	3-4 veces en la vida	5 veces o más
☐	☐	☐	☐	☐

40. ¿Te ha ocurrido alguna de las siguientes situaciones? (Elige todas OPCIONES que aplique a ti en CADA categoría)

		Sí, durante los últimos 30 días	Sí, hace más de 1 mes, pero menos de 1 año	Sí, hace más de 1 año	No
a)	Un accidente grave	☐	☐	☐	☐
b)	Una enfermedad grave	☐	☐	☐	☐
c)	Separación o divorcio de tus padres	☐	☐	☐	☐
d)	Una discusión seria con tus padres	☐	☐	☐	☐
e)	Presenciaste una pelea seria de tus padres	☐	☐	☐	☐
f)	Presenciaste violencia física en tu casa donde un adulto estaba involucrado	☐	☐	☐	☐
g)	Presenciaste violencia psicológica en tu casa donde un adulto estaba involucrado	☐	☐	☐	☐
h)	Estuviste involucrado en violencia física en tu casa en que un adulto estaba involucrado	☐	☐	☐	☐
i)	La muerte de uno de tus padres o hermano	☐	☐	☐	☐
j)	La muerte de un amigo	☐	☐	☐	☐
k)	El término de una relación con tu pololo/a	☐	☐	☐	☐
l)	Has sido rechazado/a por tus amigos	☐	☐	☐	☐
m)	La separación de un amigo/a	☐	☐	☐	☐
n)	Haber recibido excepcionalmente una mala nota	☐	☐	☐	☐
o)	Tu padre (o madre) perdió su trabajo	☐	☐	☐	☐
p)	Haber sido expulsado de la sala o haber sido enviado a inspectoría	☐	☐	☐	☐
q)	Haber sido expulsado del colegio	☐	☐	☐	☐
r)	Experimentado abuso sexual (como víctima)	☐	☐	☐	☐
s)	Experimentado abuso sexual donde un adulto dentro de la familia estaba involucrado	☐	☐	☐	☐
t)	Experimentado abuso sexual donde un adulto de fuera de la familia estaba involucrado	☐	☐	☐	☐

41. ¿Qué tan de acuerdo estas con los siguientes enunciados? (Elige UNA opción en CADA categoría)

	Totalmente de acuerdo	Algo de acuerdo	Algo en desacuerdo	Totalmente en desacuerdo
a) Siento que soy tan valioso/a como las otras personas	☐	☐	☐	☐
b) Siento que tengo muchas cualidades	☐	☐	☐	☐
c) En general tiendo a sentir que soy un fracaso	☐	☐	☐	☐
d) Soy capaz de hacer cosas tan bien como otras personas	☐	☐	☐	☐
e) Siento que no tengo muchas cosas por las cuales estar orgulloso(a)	☐	☐	☐	☐
f) Tomo una actitud positiva hacia mí mismo(a)	☐	☐	☐	☐
g) En general, estoy satisfecho(a) conmigo mismo(a)	☐	☐	☐	☐
h) Desearía tener más respeto por mí mismo(a)	☐	☐	☐	☐
i) A veces pienso que no soy bueno(a) en nada	☐	☐	☐	☐
j) Realmente me siento inútil a veces	☐	☐	☐	☐

42. ¿Qué tan de acuerdo estás con los siguientes enunciados? (Elige UNA opción en CADA categoría)

	Totalmente en desacuerdo	Algo en desacuerdo	Algo de acuerdo	Totalmente de acuerdo
a) Hay una gran cantidad de vida social en mi barrio/comunidad	☐	☐	☐	☐
b) Es bueno vivir en mi barrio/comunidad	☐	☐	☐	☐
c) En el futuro me gustaría seguir viviendo en el barrio/comunidad en que vivo actualmente	☐	☐	☐	☐
d) En el futuro me gustaría cambiarme a otra barrio/comuna/ciudad en mi país	☐	☐	☐	☐
e) En el futuro me gustaría irme a vivir al extranjero	☐	☐	☐	☐

43. Durante los últimos 12 meses, ¿tú te has...? (Elige UNA opción en CADA categoría)

	Si	No
a) ...cambiado de barrio o comunidad	☐	☐
b) ...cambiado de colegio	☐	☐

44. Durante los últimos 5 años, ¿te has... ? (Elige UNA opción en CADA categoría)

	Nunca	Una vez	Dos veces	Tres veces	Cuatro veces	Cinco veces o más
a) ... cambiado de barrio o comunidad	☐	☐	☐	☐	☐	☐
b) ... cambiado de colegio	☐	☐	☐	☐	☐	☐

45. ¿Cuán bien te representan los siguientes enunciados? (Elige UNA opción en CADA categoría)

	Me representa muy bien	Me representa bien	Me representa poco	Me representa muy poco
a) Yo creo en Dios	☐	☐	☐	☐
b) Mi fe es importante para mi	☐	☐	☐	☐
c) Yo rezo a Dios regularmente	☐	☐	☐	☐
d) Yo leo regularmente las escrituras de mi fe	☐	☐	☐	☐
e) Asisto a servicios religiosos regularmente	☐	☐	☐	☐
f) Participo regularmente de actividades religiosas en mi iglesia, distintas a las misas, cultos y otros ritos religiosos	☐	☐	☐	☐
g) Sería capaz de obtener apoyo de Dios si lo necesitara	☐	☐	☐	☐
h) He buscado apoyo de Dios cuando lo he necesitado	☐	☐	☐	☐
i Mis mejores amigos son personas religiosas	☐	☐	☐	☐
j) La mayoría de mis conocidos son personas religiosas	☐	☐	☐	☐
k) Mi madre (o adoptiva/madrastra) es religiosa	☐	☐	☐	☐
l) Mi padre (o adoptivo/padrastro) es religioso	☐	☐	☐	☐

46. Si bebes algo de las siguientes bebidas, ¿cuánto bebes cada día? (Elige UNA opción en CADA categoría)

	No la bebo	Una taza	Dos tazas	Tres tazas	Cuatro tazas	Cinco tasas	Seis o más tasas
a) Café	☐	☐	☐	☐	☐	☐	☐
b) Té	☐	☐	☐	☐	☐	☐	☐

	No la bebo	Una lata o botella mini	Dos latas o botellas mini	Tres latas o botellas mini	Cuatro latas o botellas mini	Cinco latas o botellas mini	Seis o más latas o botellas mini
c) Bebidas cola (como Coca-cola, Pepsi etc.)	☐	☐	☐	☐	☐	☐	☐
d) Bebidas energéticas que contienen cafeína (como: Red bull, Monster etc.)	☐	☐	☐	☐	☐	☐	☐

47. ¿Alguna de las siguientes personas fuma cigarrillos diariamente? (Elige UNA opción en CADA categoríay)

	No	Si	No aplica
a) Padre o apoderado	☐	☐	☐
b) Madre o apoderada	☐	☐	☐
c) Hermano(a) (uno o más)	☐	☐	☐
d) Mejor amigo(a)	☐	☐	☐

48. ¿Cuántas veces has fumado cigarrillos en tu vida? (Elige solo UNA opción)

Nunca	1-2 veces	3-5 veces	6-9 veces	10-19 veces	20-39 veces	40 veces o más
☐	☐	☐	☐	☐	☐	☐

49. ¿Cuántos cigarrillos has fumado, en promedio, durante los últimos 30 días? (Elige solo UNA opción)

☐ Nada
☐ Menos de un cigarro a la semana
☐ Menos de un cigarro al día
☐ 1-5 cigarros por día
☐ 6-10 cigarros por día
☐ 11-20 cigarros por día
☐ Más de 20 cigarros por día

50. ¿Cuántas veces, si lo has hecho, has fumado cigarros electrónicos en tu vida? (Elige solo UNA opción)

Nunca	1-2 veces	3-5 veces	6-9 veces	10-19 veces	20-39 veces	40 veces o más
☐	☐	☐	☐	☐	☐	☐

51. ¿Con qué frecuencia has fumado cigarrillos electrónicos, en promedio, <u>durante los últimos 30 días</u>? (Elige solo UNA opción)

Nunca	Menos de una vez por semana	Menos de una vez por día	1-5 veces al día	6-10 veces al día	11-20 veces al día	Más de 20 veces al día
☐	☐	☐	☐	☐	☐	☐

52. ¿Cuántas veces en tu vida has usado rapé, tabaco masticable u otros tabacos aplicables en la boca? (Elige UNA opción)

Nunca	1-2 veces	3-5 veces	6-9 veces	10-19 veces	20-39 veces	40 veces o más
☐	☐	☐	☐	☐	☐	☐

53. ¿Cuántas veces, <u>en los últimos 30 días</u>, has usado Rapé, tabaco masticable u otros tabacos aplicables en la boca? (Elige UNA opción)

Nunca	1-2 veces	3-5 veces	6-9 veces	10-19 veces	20-39 veces	40 veces o más
☐	☐	☐	☐	☐	☐	☐

54. ¿Cuántas veces has usado <u>pipa de agua / narguile</u>? (Elige UNA opción en CADA categoría)

	Nunca	1-2 veces	3-5 veces	6-9 veces	10-19 veces	20-39 veces	40 veces o más
a) En tu vida	☐	☐	☐	☐	☐	☐	☐
b) Durante los últimos 30 días	☐	☐	☐	☐	☐	☐	☐

55. ¿Cuántas veces has usado lo siguiente <u>en tu vida</u>? (Elige UNA opción en CADA categoría)

	Nunca	1-2 veces	3-5 veces	6-9 veces	10-19 veces	20-39 veces	40 veces o más
a) Ritalin, Concerta, Rubifen, Aradix, Metilfenidato, Anfetaminas (u otro medicamento para el déficit atencional) que ha sido recetado para ti	☐	☐	☐	☐	☐	☐	☐
b) Ritalin, Concerta, Rubifen, Aradix, Metilfenidato, Anfetaminas (u otro medicamento para el déficit atencional) sin receta	☐	☐	☐	☐	☐	☐	☐

56. **¿Cuántas veces has consumido alguno de los siguientes tragos <u>durante los últimos 30 días</u>?** (Elige UNA opción en CADA categoría)

	Nunca	1-2 veces	3-5 veces	6-9 veces	10-19 veces	20-39 veces	40 veces o más
a) Cerveza	☐	☐	☐	☐	☐	☐	☐
b) Alcopops o Bebidas alcohólicas suaves como "Lemon stone", "Mistral Ice", "Capel Ice" u otras	☐	☐	☐	☐	☐	☐	☐
c) Vino	☐	☐	☐	☐	☐	☐	☐
d) Destilados (pisco, ron, vodka u otro)	☐	☐	☐	☐	☐	☐	☐

57. **¿Cuántas veces has bebido alcohol de cualquier tipo?** (Elige UNA opción en CADA categoría)

	Nunca	1-2 veces	3-5 veces	6-9 veces	10-19 veces	20-39 veces	40 veces o más
a) En tu vida	☐	☐	☐	☐	☐	☐	☐
b) Durante los últimos 30 días	☐	☐	☐	☐	☐	☐	☐

58. **¿Cuántas veces te has embriagado?** (Elige UNA opción en CADA categoría)

	Nunca	1-2 veces	3-5 veces	6-9 veces	10-19 veces	20-39 veces	40 veces o más
a) En tu vida	☐	☐	☐	☐	☐	☐	☐
b) Durante los últimos 30 días	☐	☐	☐	☐	☐	☐	☐

59. **¿Cuántas veces, si lo has hecho, has bebido 4 (cuatro) o más bebidas alcohólicas (e.j. cerveza, vino, destilados, cortos) dentro del periodo de una hora o menos?** (Elige UNA opción)

Nunca	1-2 veces	3-5 veces	6-9 veces	10-19 veces	20-39 veces	40 veces o más
☐	☐	☐	☐	☐	☐	☐

60. **¿Cuántas veces, si lo has hecho, has bebido 5 (cinco) o más bebidas alcohólicas (e.j. cerveza, vino, destilados, cortos) dentro del periodo de una hora o menos?** (Elige UNA opción en CADA categoría)

Nunca	1-2 veces	3-5 veces	6-9 veces	10-19 veces	20-39 veces	40 veces o más
☐	☐	☐	☐	☐	☐	☐

61. ¿Bebes alcohol en los siguientes lugares? (Elige UNA opción en CADA categoría)

	Nunca	Pocas veces	Algunas veces	Frecuente-mente
a) En tu casa	☐	☐	☐	☐
b) En la casa de otros	☐	☐	☐	☐
c) En un centro comercial	☐	☐	☐	☐
d) Al aire libre: en la calle, en la plaza, etc.	☐	☐	☐	☐
e) En una disco o bar/pub	☐	☐	☐	☐
f) En una fiesta del colegio	☐	☐	☐	☐
g) En una fiesta universitaria	☐	☐	☐	☐
h) En un centro juvenil o clubes	☐	☐	☐	☐
i) En canchas deportivas, entrenamiento o camping	☐	☐	☐	☐
j) En otro lugar	☐	☐	☐	☐

62. ¿Cuántas veces (si alguna vez lo has hecho) has consumido alguna de las siguientes drogas?
(Elige UNA opción en CADA categoría)

	Nunca	1-2 veces	3-5 veces	6-9 veces	10-19 veces	20-39 veces	40 veces o más
a) Pastillas para dormir o tranquilizantes (SIN RECETA MÊDICA)	☐	☐	☐	☐	☐	☐	☐
b) Cannabis (Hashis o Marihuana)	☐	☐	☐	☐	☐	☐	☐
c) Anfetaminas (SIN RECETA MÊDICA)	☐	☐	☐	☐	☐	☐	☐
d) LSD (Ácido)	☐	☐	☐	☐	☐	☐	☐
e) Extasis (MDMA)	☐	☐	☐	☐	☐	☐	☐
f) Cocaina	☐	☐	☐	☐	☐	☐	☐
g) Pasta Base	☐	☐	☐	☐	☐	☐	☐
h) Relevón	☐	☐	☐	☐	☐	☐	☐
i) Hongos	☐	☐	☐	☐	☐	☐	☐
j) Inhalantes (encendedores, correctores, pegamento, desodorante ambiental u otros)	☐	☐	☐	☐	☐	☐	☐
k) Esteroides anabólicos	☐	☐	☐	☐	☐	☐	☐
l) Destilados/fermentados caseros	☐	☐	☐	☐	☐	☐	☐
m) Hierbas/drogas orgánicas (San Pedro, Ayahuasca,etc.)	☐	☐	☐	☐	☐	☐	☐
n) Gas de la risa (Oxido Nitroso)	☐	☐	☐	☐	☐	☐	☐
o) Heroina	☐	☐	☐	☐	☐	☐	☐
p) Opioides sin receta médica (morfina , codeina, fentanil, tramadol y otros)	☐	☐	☐	☐	☐	☐	☐
q) Khat (u otras catinonas) o Spice (u otros cannabinoides sintéticos)	☐	☐	☐	☐	☐	☐	☐

63. **¿A qué edad hiciste (si alguna vez las has hecho) alguna de las siguientes cosas <u>por primera vez</u>?** (Elige UNA opción en CADA categoría)

	Nunca	11 años o menos	12 años	13 años	14 años	15 o más
a) Beber alcohol	☐	☐	☐	☐	☐	☐
b) Emborracharse	☐	☐	☐	☐	☐	☐
c) Fumar cigarro	☐	☐	☐	☐	☐	☐
d) Fumar cigarro diariamente	☐	☐	☐	☐	☐	☐
e) Usar marihuana	☐	☐	☐	☐	☐	☐
f) Fumar cigarros electrónicos	☐	☐	☐	☐	☐	☐
g) Usar masticables de tabaco, rapé, u otros tabacos aplicables en la boca	☐	☐	☐	☐	☐	☐

64. **¿Cuántas veces hiciste (si alguna vez las has hecho) alguna de las siguientes cosas <u>durante los últimos 12 meses</u>?** (Elige UNA opción en CADA categoría)

	Nunca	Una vez	2-5 veces	6-9 veces	10-13 veces	14-17 veces	18 veces o más
a) Robar algo que cueste menos que 3 entradas normales de cine (aprox. $10.000)	☐	☐	☐	☐	☐	☐	☐
b) Robar algo que cueste más que 3 entradas normales de cine (aprox. $10.000)	☐	☐	☐	☐	☐	☐	☐
c) Usar violencia física para robar	☐	☐	☐	☐	☐	☐	☐
d) Entrar a una casa o auto para robar	☐	☐	☐	☐	☐	☐	☐
e) Dañar o hacer vandalismo a cosas que no son tuyas	☐	☐	☐	☐	☐	☐	☐
f) Cometer otro delito	☐	☐	☐	☐	☐	☐	☐

65. ¿Cómo reaccionarían tus padres o apoderados si hicieras alguna de las siguientes cosas? (Elige UNA opción en CADA categoría)

	Estarían totalmente en contra	Estarían muy en contra	Estarían en contra	Ellos no se preocuparían
a) Si tu fumaras cigarros	☐	☐	☐	☐
b) Si tu te emborracharas	☐	☐	☐	☐
c) Si tu fumaras marihuana	☐	☐	☐	☐
d) Si tu fumaras cigarros electrónicos	☐	☐	☐	☐
e) Si tu usaras tabaco masticable, rapé u otros tabacos aplicables en la boca	☐	☐	☐	☐

66. Por favor responde las siguientes preguntas según cómo se aplican a ti. (Elige UNA opción en CADA categoría)

	Nunca	Una vez	2-5 veces	6-9 veces	10-13 veces	14-17 veces	18 veces o más
a) ¿Has sido victima de violencia física en los últimos 12 meses?	☐	☐	☐	☐	☐	☐	☐
b) ¿Has ejercido violencia física en los últimos 12 meses?	☐	☐	☐	☐	☐	☐	☐
c) ¿Has sido victima de violencia sexual en los últimos 12 meses?	☐	☐	☐	☐	☐	☐	☐
d) ¿Has ejercido violencia sexual en los últimos 12 meses?	☐	☐	☐	☐	☐	☐	☐

67. ¿Cuántas veces hiciste alguna de las siguientes cosas (si alguna vez las has hecho), durante los últimos 12 meses?

	Nunca	Una vez	Dos veces	3 - 4 veces	5 veces o más
a) He sido parte de un grupo que se burla de alguien	☐	☐	☐	☐	☐
b) He sido parte de un grupo que daña físicamente a alguien	☐	☐	☐	☐	☐
c) He sido parte de un grupo que inicia una pelea con otro grupo	☐	☐	☐	☐	☐
d) He recibido burlas por un grupo	☐	☐	☐	☐	☐
e) He sido atacado por un grupo	☐	☐	☐	☐	☐
f) He estado en un grupo que ha sido atacado por otro grupo	☐	☐	☐	☐	☐

68. ¿Cuántas veces (si alguna vez te ha ocurrido) en tu vida, te ha ocurrido lo siguiente?

	Nunca	Una vez	Dos veces	3 - 4 veces	5 veces o más
a) Yo he ENVIADO mensajes ofensivos o desagradables a un grupo o a una persona a través de internet o por el teléfono	☐	☐	☐	☐	☐
b) Yo he RECIBIDO mensajes ofensivos o desagradables de un grupo o una persona a través de internet o por el teléfono?	☐	☐	☐	☐	☐

69. ¿Cuántas veces (si alguna vez las has hecho) has hecho alguna de las siguientes cosas, en los últimos 12 meses? (Elige UNA opción en CADA categoría)

	Nunca	Una vez	2-5 veces	6-9 veces	10-13 veces	14-17 veces	18 veces o más
a) Pegarle un combo a alguien	☐	☐	☐	☐	☐	☐	☐
b) Darle una paliza a alguien	☐	☐	☐	☐	☐	☐	☐
c) Pateado a alguien	☐	☐	☐	☐	☐	☐	☐
d) Cacheteado a alguien	☐	☐	☐	☐	☐	☐	☐
e) Tomado a alguien por el cuello	☐	☐	☐	☐	☐	☐	☐
f) Amenazado a alguien con violencia	☐	☐	☐	☐	☐	☐	☐

70. ¿Cuántos de tus amigos, crees que han hecho lo siguiente en los últimos 12 meses? (Elige UNA opción en CADA categoría)

	Nadie	Unos pocos	Algunos	Muchos	Casi todos
a) Robado algo que cuesta más de 3 entradas al cine (aprox. $10.000)	☐	☐	☐	☐	☐
b) Entrado a una casa o auto para robar	☐	☐	☐	☐	☐
c) Dañado o hecho vandalismo a cosas que no son de ellos	☐	☐	☐	☐	☐

71. Cuántos de tus amigos, crees que han hecho lo siguiente? (Elige UNA opción en CADA categoría)

	Nadie	Unos pocos	Algunos	Muchos	Casi todos
a) Fumar cigarro	☐	☐	☐	☐	☐
b) Beber alcohol (cerveza, vino, o destilados)	☐	☐	☐	☐	☐
c) Embriagarse al menos una vez al mes	☐	☐	☐	☐	☐
d) Fumar marihuana	☐	☐	☐	☐	☐
e) Buscar peleas	☐	☐	☐	☐	☐

72. Las siguientes preguntas son acerca de deportes y actividades aeróbicas (Elige UNA opción en CADA categoría)

	Casi nunca o nunca	Una vez a la semana	Dos veces a la semana	3 veces a la semana	4-6 veces a la semana	Casi todos los días
a) ¿Con qué frecuencia practicas deportes o entrenamiento físico en el colegio, fuera de las clases de educación física?	☐	☐	☐	☐	☐	☐
b) ¿Con qué frecuencia practicas deporte como miembro de un club deportivo o equipo?	☐	☐	☐	☐	☐	☐
c) ¿Con qué frecuencia haces ejercicio o practicas deporte, fuera del colegio y <u>fuera</u> de un club deportivo o equipo?	☐	☐	☐	☐	☐	☐
d) ¿Con qué frecuencia te esfuerzas físicamente hasta agotarte o sudar?	☐	☐	☐	☐	☐	☐

73. ¿Participas en alguna actividad extracurricular o recreacional organizada? (Elige solo UNA opción)

Casi nunca	Una vez a la semana	Dos veces a la semana	3 veces a la semana	4-6 veces a la semana	Casi todos los días
☐	☐	☐	☐	☐	☐

74. ¿Cuantos días de la última semana, hiciste al menos 60 minutos de ejercicio de una intensidad sufciente como para acelerar tu respiración? (Elige solo UNA opción) *puede icluir deportes, bicileta, caminata u otros, por entretención o para desplazarte de un lugar a otro.*

Casi nunca	Una vez a la semana	Dos veces a la semana	3 veces a la semana	4-6 veces a la semana	Casi todos los días
☐	☐	☐	☐	☐	☐

75. ¿Cuánto tiempo, en promedio, pasas cada día en las siguientes actividades? (Elige solo una opción)

	Casi nada	½ a 1 hora	Cerca de 1 hora	Cerca de 2 horas	Cerca de 3 horas	Cerca de 4 horas	Cerca de 5 horas	6 horas o más
a) Viendo programas, películas o videos	☐	☐	☐	☐	☐	☐	☐	☐
b) Jugando videojuegos	☐	☐	☐	☐	☐	☐	☐	☐
c) En redes sociales (e.j. Facebook, Snapchat, Messenger, Instagram, Twitter, Vine, Skype, WhatsApp, Tumblr. Etc)	☐	☐	☐	☐	☐	☐	☐	☐
c) Usando internet en otras actividades o videojuegos (e.j. leyendo, viendo noticias)	☐	☐	☐	☐	☐	☐	☐	☐

76. ¿Que tan seguido ha ocurrido lo siguiente? (Elige sólo una OPCIÓN)

	Nunca	1-2 veces	3-5 veces	6-9 veces	10-19 veces	20-39 veces	40 veces o más
a) Enviaste una foto provocativa o desnudo/a a alguien a través de internet	☐	☐	☐	☐	☐	☐	☐
b) Le pediste a alguien que te enviara una foto provocativa o desnudo/a suya a través de internet o mensajes/chat	☐	☐	☐	☐	☐	☐	☐
c) Alguien te ha pedido que envíes una foto provocativa o desnudo/a a través de internet o mensajes/chat	☐	☐	☐	☐	☐	☐	☐

77. En una escala de 1 a 5, donde 1 es "sin atracción" y 5 es "Intensa atracción". (Marca con una x la posición que más te represente)

¿Qué tan atraído/a te sientes hacia personas del sexo **opuesto?**

Sin atracción	1	2	3	4	5	Intensa atracción
☐	☐	☐	☐	☐	☐	☐

¿Qué tan atraído/a te sientes hacia personas de tu **mismo** sexo?

Sin atracción	1	2	3	4	5	Intensa atracción
☐	☐	☐	☐	☐	☐	☐

PLANET Youth
EVIDENCE BASED DRUG PREVENTION

UNIVERSIDAD DE CHILE

Código de serie de la municipalidad

Por favor pon el cuestionario en el sobre, pégalo y devuélvelo al profesor / supervisor.

Todos los cuestionarios serán destruidos luego de extraídos los datos.

Estamos muy agradecidos por tu participación.

© ICSRA 2018
